# Studying Brugada Syndrome With an SCN1B Variants in Human-Induced Pluripotent Stem Cell-Derived Cardiomyocytes

**DOI:** 10.3389/fcell.2019.00261

**Published:** 2019-11-01

**Authors:** Ibrahim El-Battrawy, Jonas Müller, Zhihan Zhao, Lukas Cyganek, Rujia Zhong, Feng Zhang, Mandy Kleinsorge, Huan Lan, Xin Li, Qiang Xu, Mengying Huang, Zhenxing Liao, Alexander Moscu-Gregor, Sebastian Albers, Hendrik Dinkel, Siegfried Lang, Sebastian Diecke, Wolfram-Hubertus Zimmermann, Jochen Utikal, Thomas Wieland, Martin Borggrefe, Xiaobo Zhou, Ibrahim Akin

**Affiliations:** ^1^First Department of Medicine, University Medical Centre Mannheim (UMM), Mannheim, Germany; ^2^DZHK (German Center for Cardiovascular Research), Partner Sites Heidelberg-Mannheim and Göttingen, Mannheim, Germany; ^3^Stem Cell Unit, Clinic for Cardiology and Pneumology, University Medical Center Göttingen, Göttingen, Germany; ^4^Key Laboratory of Medical Electrophysiology of Ministry of Education and Medical Electrophysiological, Key Laboratory of Sichuan Province, Institute of Cardiovascular Research, Southwest Medical University, Luzhou, China; ^5^Center for Human Genetics and Laboratory Medicine, Martinsried, Germany; ^6^Max Delbrück Center for Molecular Medicine, Berlin, Germany; ^7^Institute of Pharmacology and Toxicology, University of Göttingen, Göttingen, Germany; ^8^Skin Cancer Unit, German Cancer Research Center (DKFZ), Heidelberg and Department of Dermatology, Venereology and Allergology, University Medical Center Mannheim, University of Heidelberg, Mannheim, Germany; ^9^Institute of Experimental and Clinical Pharmacology and Toxicology, Medical Faculty Mannheim, University of Heidelberg, Mannheim, Germany

**Keywords:** Brugada, sudden cardiac death, channelopathy, genetic, sodium channel

## Abstract

**Background:**

Among rare channelopathies BrS patients are at high risk of sudden cardiac death (SCD). SCN5A mutations are found in a quarter of patients. Other rare gene mutations including SCN1B have been implicated to BrS. Studying the human cellular phenotype of BrS associated with rare gene mutation remains lacking.

**Objectives:**

We sought to study the cellular phenotype of BrS with the SCN1B gene variants using human-induced pluripotent stem cell (hiPSCs)–derived cardiomyocytes (hiPSC-CMs).

**Methods and Results:**

A BrS patient suffering from recurrent syncope harboring a two variants (c.629T > C and c.637C > A) in SCN1B, which encodes the function-modifying sodium channel beta1 subunit, and three independent healthy subjects were recruited and their skin biopsies were used to generate hiPSCs, which were differentiated into cardiomyocytes (hiPSC-CMs) for studying the cellular electrophysiology. A significantly reduced peak and late sodium channel current (I_Na_) and a shift of activation curve to more positive potential as well as a shift of inactivation curve to more negative potential were detected in hiPSC-CMs of the BrS patient, indicating that the SCN1B variants impact the function of sodium channels in cardiomyocytes. The reduced I_Na_ led to a reduction of amplitude (APA) and upstroke velocity (V_*max*_) of action potentials. Ajmaline, a sodium channel blocker, showed a stronger effect on APA and Vmax in BrS cells as compared to cells from healthy donors. Furthermore, carbachol was able to increase arrhythmia events and the beating frequency in BrS.

**Conclusion:**

Our hiPSC-CMs from a BrS-patient with two variants in SCN1B recapitulated some key phenotypic features of BrS and can provide a platform for studies on BrS with SCN1B variants.

## Introduction

Sudden cardiac death (SCD) is related to ventricular tachyarrhythmias. Right bundle branch block and coved ST-segment elevation in precordial leads (V1 to V3) at baseline or after using sodium channel blocking drug are diagnostic markers of Brugada syndrome (BrS). In addition, fever could potentially unmask BrS. Therefore a consensus statement of the ESC guideline 2015 recommended avoiding fever and/or infections as a trigger of BrS and/or arrhythmias (Casado-Arroyo et al., 2016). BrS patients and their families are at risk to develop malignant ventricular tachyarrhythmias and SCD (Morita et al., 2002). BrS patients may have other symptoms including recurrent syncope and atrial arrhythmias. In men <40 years old BrS is a denominator cause of SCD.

More than 300 mutations in 12 genes, mainly in SCN5A (encoding the ion channel Na_v_ 1.5), have been reported in more than a quarter of patients (Risgaard et al., 2013). The relation of further genes including SCN1B (encoding the sodium channel beta1 subunit) has been debated (Hu et al., 2009). Due to the lack of suitable human cardiomyocyte models for the majority of gene mutations, mimicking the key features of BrS, their genotype-phenotype association is controversial. Recently published data from human induced pluripotent stem cell-derived cardiomyocytes (hiPSC-CMs) from BrS patients in the presence of mutations in SCN5A demonstrated a successful establishment of a cellular BrS model and confirmed experimentally the genotype-phenotype association of SCN5A mutations versus BrS (Liang et al., 2016). In 2008, the first report about the role of SCN1B in BrS was published (Watanabe et al., 2008). Watanabe et al. recruited 326 patients suffering from BrS and conduction disease and investigated the presence of SCN1B mutations. In that study mutations in SCN5A have been excluded. Three mutations (c.259G > C, c.536G > A, c.537G > A) in SCN1B were identified in three families. A co-expression of mutant beta1 or beta1B subunits in CHO cells with Na_v_ 1.5 showed a lower sodium channel current as compared with a co-expression with wild type subunits of SCN1B. Those data suggested an important role of SCN1B for cardiac sodium channel current and an association of SCN1B mutations with BrS. But until now, cellular models of BrS with SCN1B mutations remain lacking.

In BrS patients a cardioverter defibrillator (ICD) for secondary prevention is recommended. Of note, ICD is associated with a bevy of complications. Therefore, pharmacotherapy is also required, at least for some patients. Models of BrS are warranted for drug screening and/or studying the pathomechanisms of ventricular tachyarrhythmias in BrS.

Recently published data demonstrated that hiPSC-CMs are useful for research on pathophysiology of cardiac disorders (El-Battrawy et al., 2016). Ion channel characteristics and action potential characteristics of hiPSC-CMs are comparable to human ventricular cardiomyocytes (Zhao et al., 2018). Additionally, different diseases have been studied using hiPSC-CMs, making these cells suitable for further research in cardiomyopathies and channelopathies (Moretti et al., 2010; El-Battrawy et al., 2016, 2018a,b,c,d; Liang et al., 2016; Yucel et al., 2017). Based on those reports, we aimed to study the cellular phenotype of a BrS patient in presence of a compound mutation in the SCN1B using hiPSC-CMs and provide a cellular model of BrS with SCN1B mutations for further pathophysiology and drug screening studies.

## Materials and Methods

### Ethics Statement

One BrS patient and three healthy donors were recruited for skin biopsy after obtaining a written informed consent. The Ethics Committee of the Medical Faculty Mannheim, University of Heidelberg (approval number: 2018-565N-MA) and the Ethics Committee of University Medical Center Göttingen (approval number: 10/9/15) have approved the study, which was carried out in accordance with the guidelines of Helsinki Declaration of 1975, as revised in 1983.

### DNA-Sequencing Analysis

DNA was isolated from blood lymphocytes. Ninety-eight of 107 coding exons of the genes CACNA1C, CACNB2, GPD1L, KCNE3, SCN1B, SCN3B, SCN10A, and SCN5A including exon/intron boundaries were amplified by PCR and subjected to bidirectional Sanger sequencing. Sequences were mapped against hg19 references (NM_015141.3, NM_000719.5, NM_201590.2, NM_199037.4, NM_005472.4, NM_018400.3, NM_006514.3, and NM_198056.2) via JSI Sequence Pilot for further analysis.

### Generation of Human iPS Cells

Human iPS cells (hiPSCs) were generated from primary human fibroblasts derived from skin biopsies. The BrS cell lines isBrSc2.5, isBrSc2.15, and isBrSc2.16 (UMGi128-A) from the index patient were generated in feeder free culture conditions using the integration-free CytoTune-iPS 2.0 Sendai Reprogramming Kit (Thermo Fisher Scientific) with the reprogramming factors OCT4, KLF4, SOX2, c-MYC according to the manufacturer’s instructions with modifications. The generated hiPSCs were characterized for their pluripotency and their *in vitro* differentiation potential as described (El-Battrawy et al., 2018a,b,c). The cell line from the first healthy donor (D1) was generated using lentiviral particles carrying the transactivator rtTA and an inducible polycistronic cassette containing the reprogramming factors OCT4, SOX2, KLF4 and c-MYC and was described previously (El-Battrawy et al., 2018a, b). The cell lines from the second and third healthy donor (UMGi014-B and UMGi124-A, abbreviated as D2 and D3) were generated in feeder free culture conditions using the integration-free episomal 4-in-1 CoMiP reprogramming plasmid (Addgene, #63726) with the reprogramming factors OCT4, KLF4, SOX2, c-MYC and short hairpin RNA against p53 or the integration-free CytoTune-iPS 2.0 Sendai Reprogramming Kit, respectively, and were described previously (El-Battrawy et al., 2018a, c). Newly established iPSC lines were passaged with Versene Solution (Thermo Fisher Scientific) and cultured in StemMACS iPS-Brew XF medium (Miltenyi Biotec) supplemented with 2 μM Thiazovivin (Merck Millipore) on the first day after passaging in Matrigel-coated plates for at least ten passages before being used for pluripotency characterization and differentiation experiments. Two independent cell lines from each healthy donor were used for experiments and no differences were observed between these cell lines.

For embryoid body (EB) formation, 5 × 10^4^ iPSCs together with 2.5 × 10^4^ mouse embryonic fibroblasts were plated in each well of a 96-well U-bottom plate in hES medium composed of DMEM-F12 (Thermo Fisher Scientific), 15% Knockout Serum Replacement (Thermo Fisher Scientific), 1× MEM Non-Essential Amino Acids Solution (Thermo Fisher Scientific), 50 μM β-mercaptoethanol (SERVA Electrophoresis) and 2 μM Thiazovivin, the plate was centrifuged at 250 *g* for 5 min and co-cultures were cultivated in suspension to form multicellular EB aggregates. At d2, medium was changed to differentiation medium composed of IMDM GlutaMAX (Thermo Fisher Scientific), 20% Fetal Bovine Serum (Thermo Fisher Scientific), 1× MEM Non-Essential Amino Acids Solution and 450 μM 1-Thioglycerol (Sigma-Aldrich) for further 6 days with medium change every other day. At d8, EBs were plated onto 0.1% gelatin-coated 6-well plates and cultured for up to one month in differentiation medium with medium change every other day.

### Generation of hiPSC-CMs

Frozen aliquots of hiPSCs were thawed, cultured without feeder cells and differentiated into hiPSC-CMs as described with some modifications (El-Battrawy et al., 2016; Cyganek et al., 2018). Briefly, the hiPSCs are maintained in E8 medium (STEMCELL Technologies) supplemented with human albumin and ascorbic acid. Then the directed differentiation of hiPSCs into cardiomyocytes (hiPSC-CMs) is initiated at 80–90% confluence in 24-well plates with Matrigel coated. The cardiomyocyte differentiation medium composes of RPMI 1640 with GlutaMAX and HEPES (Thermo Fisher Scientific), 0.5 mg/ml human recombinant albumin, 0.2 mg/ml L-ascorbic acid 2-phosphate and 1% Pen/Strep. For the differentiation the hiPSCs are sequentially treated with 4 μM CHIR99021 (Merck Millipore) for 48 h and then 5 μM IWP2 (Merck Millipore) for 48 h with the cardiomyocyte differentiation medium. The medium is changed to cardiomyocyte culture medium composed of RPMI 1640 with GlutaMAX, HEPES, 2% B27 (Thermo Fisher Scientific) and 1% Pen/Strep at day 8. Differentiated cells are glucose-starved and supplemented with 5 mM sodium DL-lactate to metabolically select hiPSC-CMs around day 13–15. The selected iPSC-CMs are cultured in maintenance media at least to day 40–60 for further maturation.

In our lab the differentiation of hiPS cells into cardiomyocytes (hiPSC-CMs) is regularly performed every 2 to 3 weeks. The beating hiPSC-CMs from different independent differentiations were used for studies and the data were combined. At 40 to 60 days after start of differentiation, cardiomyocytes were dissociated from 24 well plates and plated as single cells on Matrigel-coated 3.5 cm petri dishes for patch-clamp measurements and calcium transient measurements.

### Immunocytochemical Staining and Flow Cytometry of iPSCs and iPSC-CMs

Human-induced pluripotent stem cell cultures were fixed with Roti-Histofix 4% (Carl Roth) at RT for 20 min and blocked with 1% bovine serum albumin (BSA; Sigma-Aldrich) in PBS at 4°C overnight. Primary antibodies were applied in 1% BSA for 1 h at 37°C or overnight at 4°C. Secondary antibodies with minimal cross reactivity were administered in 1% BSA for 1 h at RT. For nuclear or cytosolic proteins (OCT4, SOX2, NANOG, LIN28), cells were permeabilized with 0.1% Triton-X100 (Carl Roth) in staining solution. Nuclei were stained with 4.8 μM DAPI (Thermo Fisher Scientific) for 10 min at RT. Samples were mounted in Fluoromount-G (Thermo Fisher Scientific). Antibodies and dilutions used were listed in Supplementary Table S3. Images were collected using the Axio Observer Z1 microscopy system (Carl Zeiss) with AxioCam MRm, Plan-Apochromat 20×/0.8 objective or A-Plan 10×/0.25 objective and AxioVision software.

For flow cytometry analysis, cells were dissociated into single cells, fixed in Roti-Histofix 4% at RT for 20 min and blocked with 1% BSA in PBS at 4°C for at least 2 h. iPSCs were permeabilized with 0.1% Triton-X100 in staining solution and co-incubated with fluorescence-conjugated antibodies against OCT4 and TRA-1-60 at RT for 1 h. Nuclei were co-stained with 8.1 μM Hoechst 33342 (Thermo Fisher Scientific). Subsequently, cells were analyzed using the LSRII flow cytometer (BD Biosciences) and BD FACSDiva software. Gating of cells was applied based on forward scatter area (FSC-A) and sideward scatter area (SSC-A) as well as on gating of single cells (based on DNA signal width). At least 10,000 events were analyzed per sample.

The iPSC-CMs are dissociated from 24-well plates and plated onto the culture slides (354114; Thermo Fisher Scientific, Waltham, MA, United States). All slides are fixed with 4% paraformaldehyde at room temperature for 10 min and permeabilized with 0.5% triton for 10 min. After blocking in 5% bovine serum albumin (BSA, 10270106; Thermo Fisher Scientific, Waltham, MA, United States) for 30 min, the slides are incubated in primary antibodies at 4°C, and then are incubated with the second antibodies Anti-rabbit/mouse IgG (H + L), F(ab’)2 Fragment (1:2000; Cell Signaling, United States). The primary antibodies used on hiPSC-CMs are monoclonal Anti-α-Actinin (Sarcomeric) (A7811, 1:200; Sigma-Aldrich, Merck KGaA, Darmstadt, Germany), Anti-Cardiac Troponin T antibody [1C11] (ab8295, 1:200; Abcam, Cambridge, United Kingdom) and Anti-Myl4 (A20170301415, 1:100, Cloud-Clone, United States). Three random fields per wells (at least 10 cells) are photographed and the immunofluorescence density of the positive staining per field is measured by Image J software.

### Polymerase-Chain-Reaction Assays

To quantify the steady-state mRNA expression of ion channels from the hiPSC-CMs, RNA was prepared, reverse transcribed and qPCR was performed as described (El-Battrawy et al., 2016). Gene symbols, RefSeq No. and Cat. No. of the primers used for qPCR analyses in hiPSC-CMs characterization were listed in the Supplementary Table S1. For evaluation of the characteristics of the used hiPSC lines, RT-PCR was performed as follows: Total RNA was isolated using the SV Total RNA Isolation System (Promega, #Z3105) according to manufacturer’s instructions. 100 ng RNA was used for the first-strand cDNA synthesis by using MULV Reverse Transcriptase (Thermo Fisher Scientific, #N8080018) and Oligo d(T)16 (Thermo Fisher Scientific, #N8080128). One-tenth of cDNA was used as PCR template and amplified using the GoTaq G2 DNA polymerase (Promega, #M7845) according to manufacturer’s instructions. Primer sequences, annealing temperatures and cycles used for RT-PCR analyses of the hiPSC lines are listed in the Supplementary Table S2.

### Western Blot

The hiPSC-CMs were lysed using RIPA-buffer (Sigma-Aldrich) with Protease- and Phosphatase Inhibitors (Sigma-Aldrich) added. The protein concentration was measured using the BCA-method (Thermo Scientific). For Western blot a 10% (1 mm) SDS-PAGE (Sodium dodecyl sulfate-polyacrylamide gel electrophoresis) and PVDF (polyvinylidene difluoride) membrane were used. As protein markers Precision Plus (Bio-Rad) and LMW (GE Healthcare/Amersham) were utilized. The membrane was blocked with a solution of 5% non-fat milk powder in 0.1% TBS-T buffer. As primary antibodies we used SCN5A (Abcam Cat. No ab56240), SCN1B [Abcam (Cat. No. ab107370), GAPDH (HyTest Ltd., Cat. No 5G4) and conjugated secondary antibody (Sigma-Aldrich Cat. NoA0545 (Anti-Rabbit IgG) and A3682 (Anti-Mouse IgG)]. Before taking pictures with the Fuji LAS 1000 imager, the immunoblots were enhanced with West Femto maximum sensitivity substrate (SuperSignal^®^, Thermo Scientific). The analysis of pixel density was performed using an AIDA image analyzer (v4.25).

### Patch-Clamp

After harvesting beating cardiomyocytes after 40 days standard patch-clamp recording techniques were used to measure action potential (AP) and different ion channel currents including transient outward potassium current (I_to_), slowly activating delayed rectifier potassium current (I_ks_), rapidly activating delayed rectifier potassium current (I_kr_), peak and late sodium (I_Na_), and L-type calcium (I_CaL_) channel currents in the whole-cell configuration. All studies were carried out at room temperature. From the inactivation curves of sodium channel currents, we know that at −80 mV about 50% and at −60 mV almost 100% of sodium channels are inactivated. Without current injection, most cells displayed a resting potential (RP) of around −60 mV or even less, meaning that the recorded action potentials (AP) in those cells contain no effects of sodium channel currents. APs without contribution of sodium channel currents cannot represent APs of cardiomyocytes. Therefore a constant inward current was injected to hyperpolarize cells to around −80 mV and APs were evoked by pulses with fixed frequencies. To check the real RPs in hiPSC-CMs, we recorded also RPs without current injection.

The bath solution for peak sodium current (I_Na_) measurements contained (mmol/L): 20 NaCl, 110 CsCl, 1.8 CaCl_2_, 1 MgCl_2_, 10 HEPES, 10 glucose, 0.001 nifedipine, pH 7.4 (CsOH). Microelectrodes were filled with (mmol/l): 10 NaCl, 135 CsCl, 2 CaCl_2_, 3 MgATP, 2 TEA-Cl, 5 EGTA, 10 HEPES, pH7.2 (CsOH).

The bath solution for late sodium current contained (mmol/l): 135 NaCl, 20 CsCl, 1.8 CaCl_2_, 1 MgCl_2_, 10 Hepes, 10 glucose, 0.001 nifedipine, pH 7.4 (CsOH). Microelectrodes were filled with (mmol/l): 10 NaCl, 135 CsCl, 2 CaCl_2_, 3 MgATP, 2 TEA-Cl, 5 EGTA and 10 HEPES (pH7.2 CsOH).

The bath solution for I_CaL_ recordings contained (mmol/l): 140 TEA-Cl, 5 CaCl_2_, 1 MgCl_2_, 10 Hepes, 0.01 TTX, 2 4-AP, pH 7.4 (CsOH). Microelectrodes were filled with (mmol/l): 10 NaCl, 135 CsCl, 2 CaCl_2_, 3 MgATP, 2 TEA-Cl, 5 EGTA and 10 HEPES (pH7.2 CsOH).

The bath solution (PSS) for I_to_, I_Ks_, and AP measurements contained (mmol/l): 130 NaCl, 5.9 KCl, 2.4 CaCl_2_, 1.2 MgCl_2_, 11 glucose, 10 HEPES, pH 7.4 (NaOH). For the I_to_ measurements, 10 μM nifedipine, 10 μM TTX and 1 μM E-4031 were added in the bath solution to block I_CaL_, I_Na_ and I_Kr_. 4 AP was used to identify I_to_ (the 4 AP-sensitive current). For I_Ks_ measurements 1 μM E-4031 was added. Chromanol 293B (10 μM) was used to isolate I_Ks_ from other currents. The pipette solution contained (mmol/l): 10 HEPES, 126 KCl, 6 NaCl, 1.2 MgCl_2_, 5 EGTA, 11 glucose and 1 MgATP, pH 7.2 (KOH).

To improve I_Kr_ measurements, the Cs^2+^ currents conducted by the KCNH2 (I_Kr_) channels were measured. External solution for Cs^2+^ currents (mmol/L): 140 CsCl, 2 MgCl_2_, 10 HEPES, 10 Glucose, pH = 7.4 (CsOH). Pipette solution: 140 CsCl, 2 MgCl_2_, 10 HEPES, 10 EGTA, pH = 7.2 (CsOH).

### Measurement of Intracellular Calcium Concentration

To measure the intracellular Ca^2+^ concentration ([Ca^2+^]_*i*_), cells were loaded with the fluorescent Ca^2+^-indicator Fluo-3 AM. The fluorescence of the cells was measured by using a Cairn OptoScan calcium imaging system (Cairn Research, United Kingdom). Fluorescence is excited by 488 nm and emitted at 520 nm. Changes in [Ca^2+^]_*i*_ were described by

(1) $${\left[{\mathrm{Ca}}^{2+}\right]}_{i}=k_{d}\,\left(\frac{F}{F_{\max}-F}\right)$$

where *k_d_* = dissociation constant of Fluo-3 (400 nmol/L), F = Fluo-3 fluorescence, F_max_ = Ca^2+^-saturated fluorescence obtained at the end of each experiment (Trafford et al., 1999).

### Statistical Analysis

Data were analyzed using InStat© (GraphPad, San Diego, CA, United States) and SigmaPlot 11.0 (Systat GmbH, Germany). They are presented as mean ± SEM. Kolmogorov–Smirnov test was used to find out if parametric or non-parametric tests are required. One-way ANOVA with Bonferroni post-test was indicated in comparisons of parametric data, whereas the Kruskal–Wallis test with Dunn’s multiple comparisons post-test was indicated for non-parametric data. Student’s t-test was used for comparison of variables with normal distribution. Mann–Whitney *U*-test was used for comparison of variables with non-normal distributions, respectively. Paired *t*-test was used for qualified data comparison (before and after application of a drug). Two-tailed *p* < 0.05 was considered significant.

## Results

### Characterization of Patient Specific hiPSCs and hiPSC-CMs

**FIGURE 1:**
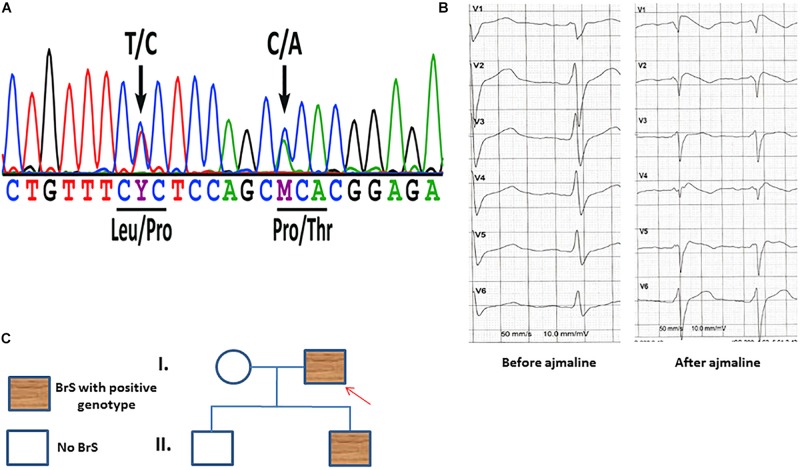
**(A)** Sanger sequencing of patient-derived cells confirmed the presence of the SCN1B variants c.629T > C/p.L210P and c.637C > A/p.P213T. **(B)** An ECG of the patient before and after receiving ajmaline (1 mg/kg) presents typical BrS changes. **(C)** A family pedigree presents the affected patient and his affected son. Both persons have the same affected gene mutations in SCN1B as described in panel **(A)**.

A 48 years old male patient with syncope at resting situation, carrying two variants (c.629T > C/p.L210P and c.637C > A/p.P213T) in SCN1B (Figure 1A), was included in our study. BrS ECG was unmasked after using ajmaline as a diagnostic challenge (Figure 1B). The family pedigree is illustrated in Figure 1C. A cascade family screening was carried out by our team excluding a structural cardiac disease and identified BrS in one son of the patient. BrS was diagnosed in his son using ajmaline to unmask BrS ECG. His son was asymptomatic. No history of sudden cardiac death was documented in the whole family. No information of other relatives or family members was available.

**FIGURE 2:**
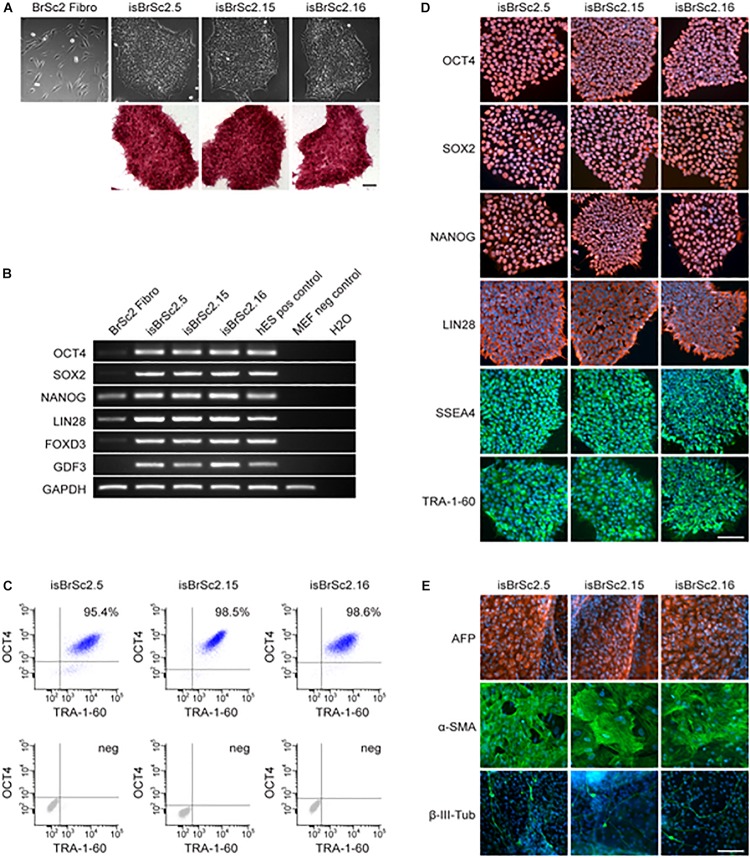
**(A)** The generated iPSC lines isBrSc2.5, isBrSc2.15, and isBrSc2.16 derived from skin fibroblasts of the BrS patient (BrSc2 Fibro) display a typical morphology for human pluripotent stem cells (upper panel) and are positive for alkaline phosphatase as being upregulated in pluripotent stem cells (lower panel). Scale bar: 100 μm. **(B)** In comparison to donor’s fibroblasts, generated iPSC lines show expression of pluripotency markers OCT4, SOX2, NANOG, LIN28, FOXD3, and GDF3 at mRNA level proven by RT-PCR. Human embryonic stem cells (hESCs) were used as positive control, mouse embryonic fibroblasts (MEFs) were used as negative control. **(C)** Flow cytometry analysis of pluripotency markers OCT4 and TRA-1-60 reveals a homogeneous population of pluripotent cells in generated iPSC lines. **(D)** Generated iPSC lines express pluripotency markers OCT4, SOX2, NANOG, LIN28, SSEA4 and TRA-1-60 as shown by immunofluorescence staining. Nuclei are co-stained with DAPI. Scale bar: 100 μm. **(E)** Spontaneous differentiation potential of generated iPSC lines was analyzed by embryoid body formation. Immunocytochemical staining of spontaneously differentiated iPSC lines shows expression of endodermal marker AFP, mesodermal-specific α-SMA and ectodermal βIII-tubulin. Nuclei are co-stained with DAPI. Scale bar: 100 μm.

Dermal skin fibroblasts of the patient were successfully reprogrammed into iPSCs and three independent patient-specific iPSC lines were characterized for pluripotency. Control iPSC lines D1-D3 have been previously studied and deeply characterized in our previous studies (El-Battrawy et al., 2016, 2018a,b,c). The hiPSCs from the BrS-patient displayed characteristic human embryonic stem cell (ESC) morphology and expression of pluripotency markers (Figures 2A–D and Supplementary Figure S1A). Additionally, spontaneous differentiation of the generated hiPSC lines via embryoid bodies confirmed expression of germ layer-specific genes from all three germ layers (Figure 2E). The presence of the two SCN1B variants as shown in Figure 1A was verified in all characterized hiPSC lines from the patient (data not shown).

Three clones of hiPSCs from the BrS-patient (BrS) and control lines (D1, D2 and D3) were differentiated into functional hiPSC-CMs in feeder-free culture conditions for subsequent functional analyses. Beating cardiomyocytes were observed 8–12 days after starting the differentiation. The mRNA level of cTnT was similarly increased over time in all cell lines (Supplementary Figure S1B). Cardio-specific markers including α-actinin, Myl4 and cTnT were detected by immunostaining (Supplementary Figure S1C) as published in other papers (El-Battrawy et al., 2018a, c).

### Changes in Ion Channel Expression in hiPSC-CMs From the BrS-Patient

The qPCR analysis was used for investigating the mRNA expression levels of different ion channels and some receptors. BrS cells showed that the mRNA levels of the ion channel SCN5A and ß2-adrenoceptor were increased compared to donor cells, but the mRNA expressions of CACNA1C, KCNJ2, KCNH2, SCN1B and SCN3B as well as ß1-adrenoceptor were lower than in donor cells (Supplementary Figures S1D,E). Western blot analysis showed a similar protein level of SCN5A but a reduced SCN1B level in BrS cells compared to healthy donor cells (Supplementary Figures S2A,B).

### Changes of I_Na_ in hiPSC-CMs From the BrS-Patient

**FIGURE 3:**
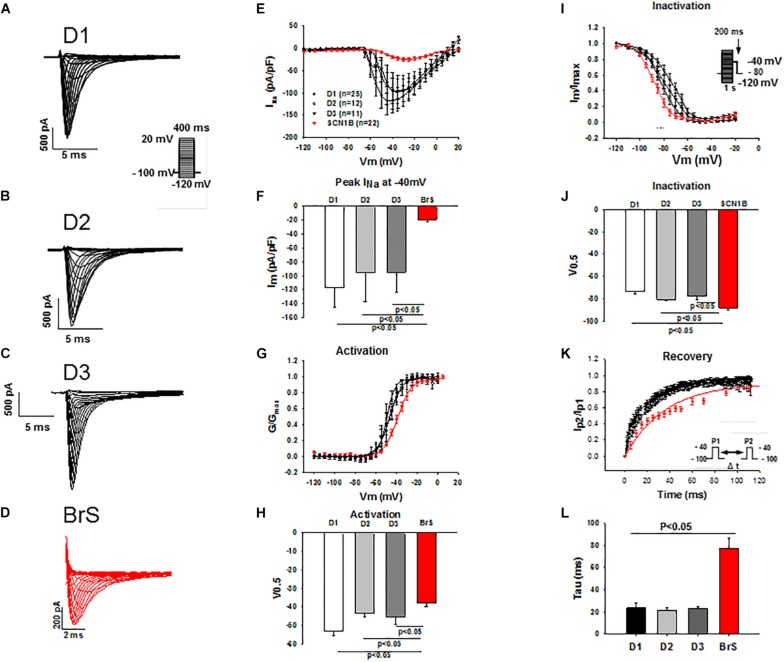
Peak I_Na_ was reduced in hiPSC-CMs from the BrS-patient. Peak I_Na_ was recorded with the protocol shown in panel A (inset) in hiPSC-CMs from donors (D1, D2, D3) and the BrS patient. The measured peak currents were plotted against voltages to obtain the current-voltage relationship (I-V) curves. I_Na_ was divided by the driving force (V-Vres, where V presents the voltage at each step, Vres represents the reverse potential for Na^+^ current) to obtain the conductance (G), which is in turn normalized to maximum (G/G_max_) and plotted against voltages to obtain the activation curves. For assessing the inactivation of sodium channels I_Na_ was recorded with the protocol indicated in I (inset) and the inactivation curves were obtained. Both activation and inactivation curves were fitted by Boltzmann equation to get the values of 50% activation or inactivation (V0,5) of sodium channels. For assessing the recovery of the channel I_Na_ was recorded with the double-pulse protocol (inset in panel **K**). The currents evoked by the second pulse were normalized to that evoked by the first pulse and then plotted against the time intervals between the two pulses. The recovery curves were fitted by single exponential equation to get the time constant (tau). **(A–D)** Representative traces of I_Na_ in hiPSC-CMs from healthy donors and the BrS patient. **(E,F)** I-V curves and I_Na_ at –40 mV showing a reduced peak I_Na_ in hiPSC-CMs from donors and the BrS patient. **(G,H)** Activation curves and the voltage values at 50% activation (V0.5). **(I,J)** Inactivation curves and the voltage values at 50% inactivation (V0.5). **(K,L)** Curves and tau values of recovery from inactivation. n, number of cells.

Peak and late sodium current I_Na_ were significantly reduced in BrS cardiomyocytes as compared to control cells (peak I_Na_, BrS −19.3 ± 3.7 pA/pF versus D1 −116.9 ± 28.7 pA/pF, D2 −95.4 ± 41.6pA/pF and D3 −94.7 ± 28.3 pA/pF; late I_Na_, BrS −0.2 ± 0.03pA/pF versus D1 −0.4 ± 0.06 pA/pF, D2 −0.4 ± 0.08 pA/pF and D3 −0.3 ± 0.06pA/pF; Figure 3 and Supplementary Figures S2C,D). While the activation curves of I_Na_ of BrS were shifted to more positive potentials (V0.5 BrS −37.8 ± 1.8 mV versus D1 −53.2 ± 2.1, D2 −43.3 ± 2.1, and D3 −45.1 ± 4.1 mV; Figures 3G,H), the inactivation curve was significantly shifted to more negative potentials (V0.5, BrS −88.1 ± 1.8 mV versus D1 −73.7 ± 2.3, D2 −80.6 ± 1.5, and D3 −77.9 ± 2.6 mV; Figures 3I,J) and the recovery of I_Na_ from inactivation was conspicuously decelerated in BrS cardiomyocytes (Figures 3K,L).

### Changes of L-Type Calcium Channel Current (I_Ca–L_) in hiPSC-CMs From the BrS-Patient

The peak I_Ca–L_ in BrS cardiomyocytes was similar to that in healthy cardiomyocytes (BrS, −7.4 ± 2.4pA/pF; D1, −8.0 ± 1.4 pA/pF; D2, −8.4 ± 1.9 pA/pF; D3, 6.5 ± 1.3 pA/pF; Supplementary Figures S3A–C), which is consistent with the findings that activation and inactivation were not changed (Supplementary Figures S3D–G). However, recovery from inactivation was conspicuously decelerated in BrS cardiomyocytes as compared to healthy donor cells (Supplementary Figures S3H,I).

In addition, by measuring the Ca^2+^ transients the intracellular calcium concentration and calcium homeostasis was examined. The systolic and diastolic concentrations in BrS cells were similar to that in healthy cardiomyocytes (Supplementary Figure S4).

### Changes of K^+^ Channel Currents in hiPSC-CMs From the BrS-Patient

K^+^ channel currents are debated to contribute to arrhythmogenesis in BrS. We studied the major K^+^ channel current contributors in cardiomyocytes, the transient outward current (I_to_), the rapidly activating delayed rectifier potassium current (I_Kr_) and slowly activating delayed rectifier potassium current (I_Ks_) using specific blockers as described previously (El-Battrawy et al., 2018c). The different K^+^ currents in BrS- and donor-cells were identified using 4-AP for I_to_, E-4031 for I_Kr_ and chromanol 293B for I_Ks_. Although the I_to_ was not changed, both the I_Ks_ (BrS 0.1 ± 0.09 pA/pF versus D1 0.7 ± 0.2 pA/pF and D2 0.7 ± 0.3 pA/pF) and the I_Kr_ (BrS 1.1 ± 0.2 pA/pF versus D1 2.5 ± 0.25 pA/pF and D2 2.2 ± 0.4 pA/pF) were significantly reduced in BrS cells as compared to healthy donor cells (Supplementary Figure S5).

### Action Potential in hiPSC-CMs

**FIGURE 4:**
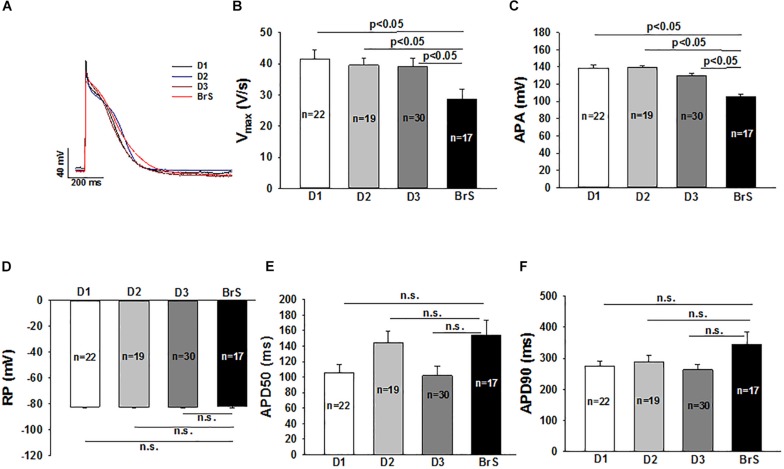
Changes of action potential in hiPSC-CMs from the BrS patient. Action potentials (AP) were evoked by pulses of 1 nA for 3 ms at 1 Hz in current clamp mode. When APs reached steady state, 10 sequential APs were recorded and mean values were calculated for each cell. All the parameters of APs were compared between cells from donors (D1, D2, D3) and the BrS patient. **(A)** Representative traces of APs in donor cells (D1, D2, and D3) and BrS cells. **(B)** Mean values of maximal depolarization velocity (Vmax) of action potentials. **(C)** Mean values of action potential amplitude (APA). **(D)** Mean values of resting potentials (RP). **(E)** Mean values of action potential duration at 50% repolarization (APD50). **(F)** Mean values of action potential duration at 90% repolarization (APD90). n, number of cells; n.s., not statistically significant (*p* > 0.05).

Action potential characterizations at 1 Hz are outlined in Figure 4. Consistent with the reduced peak I_Na_, a major contributor of phase 0 of action potential, the action potential amplitude (APA) and maximum depolarization velocity (Vmax) were significantly reduced in BrS as compared to healthy donors (Figures 4A–C). The other action potential characteristics including the resting potential (RP), the action potential duration at 50% repolarization (APD 50) and action potential duration at 90% repolarization (APD 90) were similar in the measured hiPSC-CMs from the patient and healthy donors.

### Differential Effects of Ajmaline on Action Potentials in Control and BrS Cells

**FIGURE 5:**
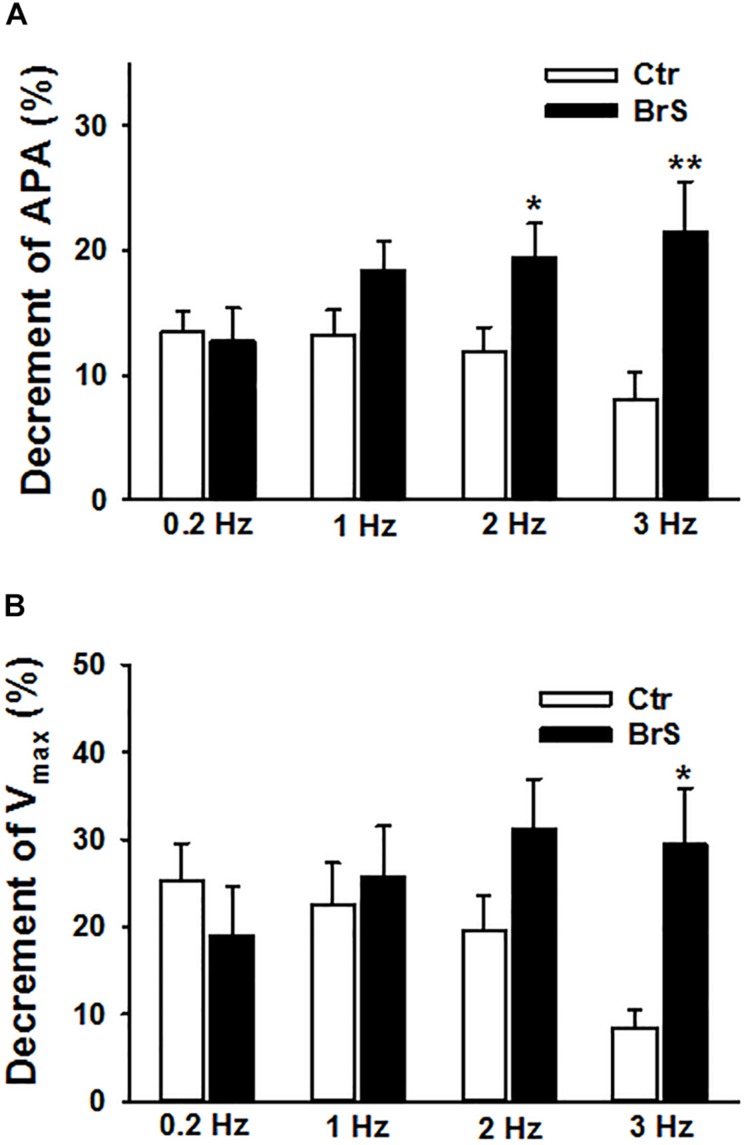
Enhanced effects of ajmaline on action potentials in hiPSC-CMs from the BrS patient. Different frequencies of stimulations were used to evoke APs. Effects of ajmaline (30 μM) on AP parameters were assessed in BrS- and donor-cells. The percent inhibition of APA and Vmax was obtained by: % inhibition = (Vctr–Vdrug)/Vctr × 100, where Vctr is the value before drug application and Vdrug is the value after drug application. **(A)** Percent inhibition of action potential amplitude (APA) by ajmaline in BrS- and donor-cells. **(B)** Percent inhibition of maximal depolarization velocity (Vmax) of action potentials by ajmaline in BrS- and donor-cells. In each, the BrS and control (D3) group, 12 cells were measured ^∗^*p* < 0.05; ^∗∗^*p* < 0.01.

To check whether ajmaline, a sodium channel current blocker that is frequently used to unmask the phenotypic changes in ECG of BrS-patients, implicates any changes in action potential characterization, 30 μM ajmaline were used to test its effect on APs at different frequencies. APs in same cells were measured sequentially from 0.2 to 3 Hz (0.2, 1, 2, 3 Hz) before and after ajmaline administration. Ajmaline reduced APA and Vmax in both donor and BrS cells. However, the effects of ajmaline in BrS cells is stronger than that in donor cells, especially at high frequencies (Figure 5), indicating a higher sensibility of BrS cells to ajmaline application.

### Arrhythmogenity of BrS Cardiomyocytes

**FIGURE 6:**
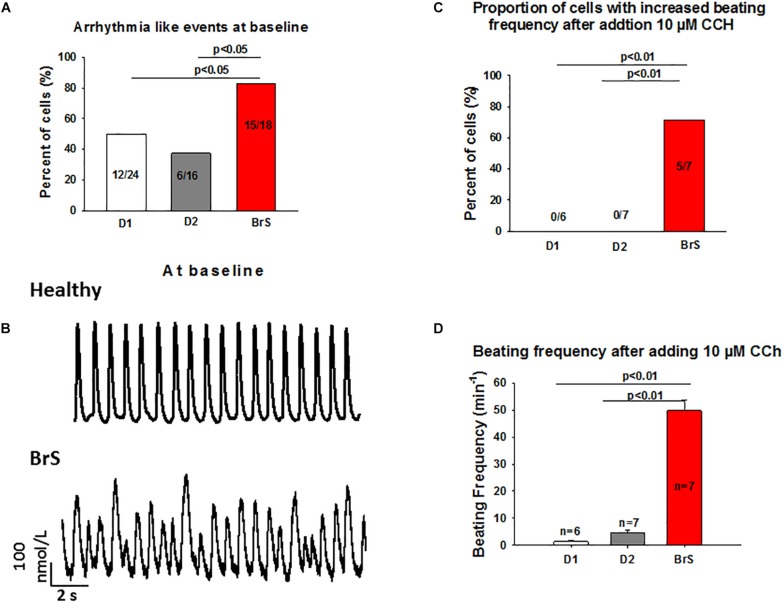
Increased frequency and arrhythmia-like events in BrS-cardiomyocytes. Single cell calcium transients were recorded in spontaneously beating cells with and without challenge by 10 μM carbachol. The rhythm (frequency, regularity, EAD- and DAD-like triggered activities) of beating cells were assessed and compared between BrS- and donor-cells. **(A)** Proportions of cells showing arrhythmia events at baseline. **(B)** Representative Ca^2+^ transients in a donor and a BrS at baseline. **(C)** Proportions of cells showing CCh-induced enhancement of beating frequency. **(D)** Beating frequencies of cells in the presence of 10 μM CCh showing CCh-induced increase in beating frequency. Values given are mean ± SEM; n, number of cells.

**FIGURE 7:**
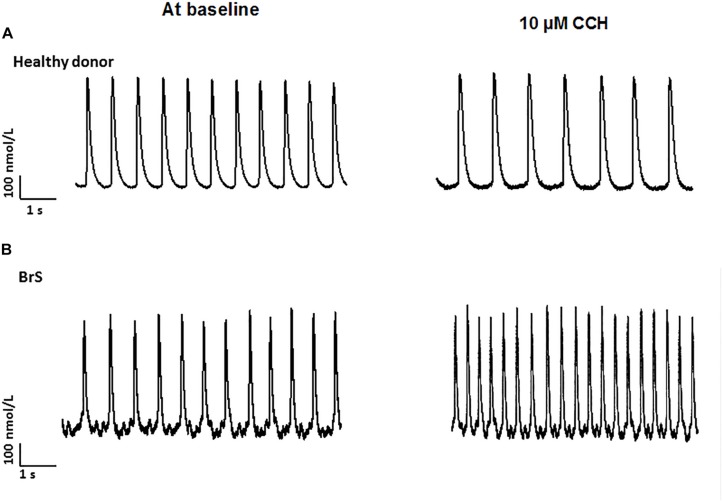
Increased frequency by carbachol in BrS-cardiomyocytes. Single cell calcium transients were recorded in spontaneously beating cells with and without challenge by 10 μM carbachol. The rhythm of beating cells were assessed and compared between BrS- and donor-cells. **(A)** Representative Ca^2+^ transients in a donor (D1) cell in absence (at baseline) and presence of carbachol (CCh). **(B)** Representative Ca^2+^ transients in a BrS cell in absence (at baseline) and presence of carbachol (CCh).

To check whether arrhythmia like events existed in BrS cardiomyocytes, spontaneous beating cells were measured at baseline and after addition of carbachol. Indeed, at baseline arrhythmia like events occurred more frequently in BrS cells than in donor cells (BrS: 11/13, D1: 12/24, D2: 6/16; *p* < 0.05; Figures 6A,B). Interestingly, after adding carbachol, a parasympathetic stimulator, donor cells showed a reduction of the beating frequency, whereas the BrS cells showed an increase in the beating frequency with arrhythmia-like events (Figures 6C,D, 7A,B). These data are consistent with the high risk of ventricular tachyarrhythmias observed in BrS patients at resting situations.

## Discussion

We report here the first model of patient specific induced pluripotent stem cell derived cardiomyocytes from a patient carrying two variants in the SCN1B gene with BrS diagnosed by ajmaline administration. We found in BrS-hiPSC-CMs: (i) The variants in SCN1B (c.629T > C/p.L210P and c.637C > A/p.P213T) led to a loss of function of sodium channels showing a significant reduction of APA and Vmax as well as sodium channel currents resulted from reduction of activation and enhancement of inactivation as well as decelerated recovery from inactivation of sodium channels; (ii) BrS-hiPSC-CMs displayed higher sensitivity responding to ajmaline and carbachol showing enhanced changes of action potential characteristics and increased arrhythmia events.

It is known that mutated SCN5A is a major contributor of BrS. However, only 20–30% of BrS patients present mutations of SCN5A. Recently published data reported that other mutations, including SCN1B are also associated with the clinical phenotype of BrS (Watanabe et al., 2008; Hu et al., 2012). Additionally, variants in SCN1B have been also related to atrial fibrillation and cardiac conduction dysfunction. But their roles in arrhythmogenesis have not been investigated yet. In the present study, we identified a genetic variant in SCN1B (c.629T > C and c.637C > A) in a family with BrS. The c.629T > C variant has been related to BrS (Ricci et al., 2014), but the genotype-phenotype correlation has not been experimentally confirmed. Whether the other one (c.637C > A) is relating to BrS is not known. The variant NM_199037.4:c.629T > C (dbSNP: rs55742440) is expected to result in the substitution of leucine to proline at amino acid position 210 (Leu210Pro) of the ß1B subunit of various sodium channels including Nav1.5. The variant Leu210Pro was observed in 78326 out of 207962 (37.66%) alleles from individuals of various ancestry in the Genome Aggregation Database (Lek et al., 2016). Due to the high allele frequency and the high number of homozygotes carrying this variant in population (Exac browser) L210P is evaluated as “benign”^1^. The variant NM_199037.4:c.637C > A (dbSNP: rs766373298) is expected to result in the substitution of proline to threonine at codon 213 (Pro213Thr). The variant Pro213Thr was observed in 2 out of 79668 (0.003%) alleles from individuals of European (Non-Finnish) ancestry and in 9 out of 17376 (0,052%) alleles from individuals of African ancestry (Lek et al., 2016). It is evaluated as “likely benign” in ClinVar^2^. Taking together, both variants are not pathogenic. Then an important point to be addressed is whether the two variants in SCN1B gene contributed to the occurrence of arrhythmias in the BrS-patient.

It should be noted that the patient recruited for this study carries the two variants in the same gene SCN1B and suffered from recurrent syncope. BrS has been identified by investigating family members using gene testing and pharmacological study with ajmaline. The hiPSC-CMs generated from the patient displayed reduced peak I_Na_, reduced V_max_ and increased arrhythmia like events, key features of BrS on a cellular level. These data indicate that the two variants of Leu210Pro and Pro213Thr in the SCN1B gene play probably an important role for the pathogenesis of BrS in that patient, which is inconsistent with aforementioned evaluations. The reason for the disparity could be: (1) Some gene variants are not pathogenic alone, but a co-factor can enhance their pathogenicity; (2) Single variant and multiple variants, especially in the same gene, may exert different effects. It is well-known that only a small part of BrS-patients have been proved to carry some gene mutations that may be linked to the disease, suggesting involvement of other unknown factors. Even in BrS-patients with known pathogenic gene mutations, arrhythmias occur only under certain conditions, suggesting involvement of some co-factor effects. It could be possible that some gene mutations are alone “benign,” but when some co-factor effects appear, their effects (like reduced I_Na_) may be enhanced and may cause disease. Furthermore, if a single variant is “benign,” it does not mean that a combination of two or more variants is also “benign.” It could be possible that two or more variants induce changes in a protein other than that induced by a single variant. Our data showing reduced I_Na_ and SCN1B expression in hiPSC-CMs from the BrS-patient indicates that the two variants in SCN1B gene influenced the protein expression and sodium channel gating, which could be at least a mechanism for the arrhythmogenesis in BrS. Moreover, the L-type calcium channel (I_Ca–L_) and some potassium channel (I_Kr_ and I_Ks_) currents in iPSC-CMs from the patient were also reduced, which resulted probably from secondary changes induced by the SCN1B variants and could contribute to the occurrence of arrhythmias in BrS. Not only changes in channel currents but also in the expression levels of channels (CACNA1C, KCNJ2, KCNH2, SCN1B, and SCN3B) were observed (Supplementary Figure S1D), although these results except for the SCN1B data should be further confirmed at protein level. The changes in the expression level of multiple ion channels indicated the existence of ion channel remodeling in cardiomyocytes carrying the two variants in SCN1B gene.

Furthermore, the L-type calcium channel (I_Ca–L_) and some potassium channel (I_Kr_ and I_Ks_) currents in iPSC-CMs from the patient were also reduced, which resulted probably from secondary changes induced by the SCN1B variants and could contribute to the occurrence of arrhythmias in BrS.

It has been demonstrated that hiPSC-CMs can model BrS with SCN5A mutations (Liang et al., 2016). Due to the lack of studies on cellular characteristics of BrS patients with SCN1B mutations using human cardiomyocytes, we aimed to investigate the cellular characteristics of a BrS patient carrying variants in the SCN1B gene by using hiPSC-CMs. The SCN1B gene encodes a ß-subunit of the sodium channel (termed Na_v_β1B) and it can also influence gating of the pore-forming a-subunit of sodium channels. It has been demonstrated that SCN1B can enhance the sodium channel currents conducted by the SCN5A channel (a-subunit). In the current study, the peak sodium channel current and the late sodium channel current, mediated mainly by SCN5A, were significantly decreased, resulting from changed gating kinetics. Given that mutations in SCN5A in our patient have been excluded, these changes are caused probably by the two variants in SCN1B. Previously published data have pointed to a possible interaction between SCN5A and SCN1B by showing co-immunoprecipitation of SCN5A and SCN1B in HEK cells (Hu et al., 2012). This may help to understand our data from the current study showing that the variants in SCN1B changed the gating and led to a reduction of function of SCN5A channels in BrS-hiPSC-CMs.

Besides, Hu et al. (2012) showed an interaction of SCN1B and KCND3, a contributor of the I_to_. Unfortunately, the present study did not detect significant changes in I_to_, although the I_kr_ and I_ks_ were significantly reduced.

Potassium current dysfunctions have been reported in BrS related to the KCNE3 gene. In our patient, mutations in potassium genes related to BrS were excluded. An imbalance of I_Na_ and I_to_ (Vatta et al., 2002) may lead to heterogeneities of action potential characteristics and may develop spatial dispersion of repolarization as denominator for ventricular tachycardia. In the current study, although we did not observe enhanced outward currents including the I_to_, we did detect the reduction of I_Na_, which may present the important phenotypic changes in BrS. The reduced I_Na_ and V_max_ can reduce propagation of excitation, in agreement with the conduction defect in BrS. In our study, we did not observe significant changes of APD. This might be explained by the counteracting effects of reduced inward current (late I_Na_) and outward currents (I_kr_, I_Ks_) on the APD. In this and aforementioned studies, no APs with spike and dome were detected probably because I_to_ was not enhanced and spike and dome are related to gradient difference between the epicard and endocard, which cannot be modeled using hiPSC-CMs.

A further interesting finding of the present study is the effect of ajmaline, which blocks sodium channel and used to unmask BrS as a diagnostic challenge. Ajmaline has shown an effect in both donor and BrS cells on APA and V_max_. However, this effect was stronger in BrS cardiomyocytes. This might confirm the higher sensitivity of BrS patients with variants in SCN1B to sodium channel blockers.

Finally, another interesting point of the study is the effect of the parasympathetic stimulator carbachol (CCh), which showed an opposite result as compared with healthy donors. While in healthy donor cells after adding CCh the beating frequency was reduced, in BrS cardiomyocytes the beating frequency was significantly increased with arrhythmic like events, which may reflect the induction of ventricular tachyarrhythmias in BrS cardiomyocytes at resting situation. Although carbachol mainly acts on muscarine receptors (M1-2), it affects also beta-adrenergic receptors causing a loss of receptors availability (Limas and Limas, 1985). Both effects should reduce the beating frequency of cardiomyocytes, which was indeed observed in donor cells challenged by CCh. Given that M3 receptors are also expressed in hiPSC-CMs (Supplementary Figure S1E), we speculate that CCh may stimulate M3 receptor and leads to enhanced Ca^2+^ release or Ca^2+^ oscillations through Gq-PLC-signaling. This may help understand why CCh increased the frequency and abnormal Ca^2+^-transients, but cannot explain why these effects of CCh were observed only in BrS cells because no difference of expression levels of M-receptors including M3, M3 and M4 was detected among donor and BrS cells. The observed reduction of ß1 and elevation of ß2 adrenoceptor expression cannot explain the effect of CCh either. Therefore, how CCh increased the beating frequency with arrhythmia-like events in BrS-hiPSC-CMs needs to be clarified.

## Conclusion

Our data demonstrated that the hiPSC-CMs from a BrS-patient with SCN1B gene variants recapitulated the main features of BrS, showing reduced I_Na_, APA and Vmax, an increased occurrence of arrhythmia-like events and enhanced sensitivity to ajmaline challenge. The hiPSC-CM model may provide a suitable platform for studying BrS and may be helpful for drug screening.

## Study Limitations

Differences among individuals of healthy probands cannot be ruled out. Only one patient was recruited for this study because we do not have a second patient with the same variants and it should be very difficult to find another patient with same variants in the same gene. This limitation cannot be overcome before other patients with the same variants in SCN1B are available. Anyway, the study is clinical relevant, at least for personal medicine, which could be investigated in future studies. HiPSC-CMs are differing from adult human cardiomyocytes despite distinct similarities in their physiological properties. The immaturity is a limitation of hiPSC-CMs for mimicking the mature cardiomyocytes in patients. However, most ion channels in hiPSC-CMs including their electrical properties are similar to those in mature cardiomyocytes. Therefore, it provides a good opportunity to study ion channel functions under physiological or pathological conditions. Furthermore, many intrinsic mechanistic factors like nerve and hormone regulations and cell to cell interaction are not considered, which might be important in BrS. Finally, the spike and dome shape in APs may contribute to forming the ECG-phenotype of BrS. The lack of APs with spike and dome of the present study indicates a failure of hiPSC-CMs to recapitulate this feature of BrS. However, it recapitulated other important features of BrS including loss of function of SCN5A, reduced peak I_Na_ and arrhythmogenesis.

## Data Availability Statement

All datasets generated for this study are included in the article/Supplementary Material.

## Ethics Statement

The studies involving human participants were reviewed and approved by the University Medical Center Mannheim, Ethics Commission II, University of Heidelberg. The patients/participants provided their written informed consent to participate in this study.

## Author Contributions

IE-B, JM, MB, XZ, and IA wrote the first draft of the manuscript. SL, SD, W-HZ, JU, and TW supervised the experiments. ZZ, LC, RZ, FZ, MK, HL, XL, QX, MH, ZL, AM-G, SA, and HD analyzed the data.

## Conflict of Interest

The authors declare that the research was conducted in the absence of any commercial or financial relationships that could be construed as a potential conflict of interest.

## Footnotes

1 http://exac.broadinstitute.org/variant/19-35524824-T-C

2 https://www.ncbi.nlm.nih.gov/clinvar/variation/190846/
