# Supplementary material for: Studying Brugada Syndrome With an SCN1B Variants in Human-Induced Pluripotent Stem Cell-Derived Cardiomyocytes
Source: Front Cell Dev Biol. 2019 Nov 1;7:261. doi: 10.3389/fcell.2019.00261 (PMC6839339; doi:10.3389/fcell.2019.00261)
Supplement: Supplementary file 1 [file Data_Sheet_1.doc]

**Supplementary information**

# Studying Brugada Syndrome with an SCN1B Variants in Human-Induced Pluripotent Stem Cell-Derived Cardiomyocytes

**Running title: SCN1B variants induced cellular phenotype**

Ibrahim El-Battrawy^1,2^**^*^**, Jonas Müller^1,2*^, Zhihan Zhao^1,2^, Lukas Cyganek^2,3^, Rujia Zhong^1^, Feng Zhang^1^, Mandy Kleinsorge^2,3^, Huan Lan^1,2,4^, Xin Li^1^, Qiang Xu^1^, Mengying Huang^1^, Zhenxing Liao^1^, Alexander Moscu-Gregor^5^, Sebastian Albers^1,2^, Hendrik Dinkel^1^, Siegfried Lang^1,2^, Sebastian Diecke^6^, Wolfram-Hubertus Zimmermann^2,7^, Jochen Utikal^2,8^, Thomas Wieland^2,9^, Martin Borggrefe^1,2^, Xiaobo Zhou^1,2,4^, Ibrahim Akin^1,2^

^1^First Department of Medicine, University Medical Centre Mannheim (UMM), University of Heidelberg, Mannheim, Germany

^2^DZHK (German Center for Cardiovascular Research), Partner Site, Heidelberg-Mannheim and Göttingen, Germany

^3^Stem Cell Unit, Clinic for Cardiology and Pneumology, University Medical Center Göttingen, Göttingen, Germany

**^4^Key Laboratory of Medical Electrophysiology of Ministry of Education and Medical Electrophysiological Key Laboratory of Sichuan Province,** Institute of Cardiovascular Research, Southwest Medical University, Luzhou, Sichuan 646000, China

**^5^**Center for Human Genetics and Laboratory Medicine, Martinsried, Germany

^6^Max Delbrück Center for Molecular Medicine, Berlin, Germany

^7^Institute of Pharmacology and Toxicology, University of Göttingen, Göttingen, Germany

^8^Skin Cancer Unit, German Cancer Research Center (DKFZ), Heidelberg and Department of Dermatology, Venereology and Allergology, University Medical Center Mannheim, University of Heidelberg, Mannheim, Germany

^9^Institute of Experimental and Clinical Pharmacology and Toxicology, Medical Faculty Mannheim, University of Heidelberg, Mannheim, Germany

* contributed equally

Conflict of Interest: none

**Address for correspondence**: Xiaobo Zhou, MD, First Department of Medicine, University Medical Centre Mannheim, Theodor-Kutzer-Ufer 1-3, 68167 Mannheim, Germany. Phone: 0049-621-383-1448. Fax: 0049-621-383-1474. E-mail: Xiaobo.zhou@medma.uni-heidelberg.de

**Table S1**

Gene symbols, RefSeq No. and Cat. No. of the primers used for qPCR analyses in

hiPSC-CMs characterization.

| **Gene symbol** | **RefSeq No.** | **Cat. No. Primers** |
| --- | --- | --- |
| ADRB1 | NM_000684 | PPH02091B |
| ADRB2 | NM_000024 | PPH01856E |
| CACNA1C (L-type Ca2+ channel) | NM_000719 | PPH01378G |
| CACNb2 | NM_000724 | QT00011256 |
| CHRM2 | NM_000739 | PPH02690C |
| CHRM3 | NM_000740  NM_000740 | PPH02721A |
| CHRM4 | NM_000741 | PPH02691A |
| KCND3 (Ito, Kv4.3) | NM_004980 | PPH06923A |
| KCNH2 (IKr, Kv11.1) | NM_000238 | PPH01660A |
| KCNJ2 | NM_000891 | PPH01618E |
| KCNQ1 (I_Ks_, Kv7.1) | NM_000218 | PPH01419A |
| KCNIP2 | NM_014591 | QT00016254 |
| POU5F1 | NM_002701.5 | PPH02394E |
| SCN1B | NM_001037 | QT00066080 |
| SCN3B | NM_018400 | PPH07274A |
| SCN5A (Na+ channel, Nav1.5) | NM_000335 | PPH01671F |
| SCN10A (Na+ channel, Nav1.8) | NM_006514 | PPH15064A |
| TNNT2 | NM_000364 | PPH02619A |

RefSeq No. : GenBank NCBI Reference Sequences

Cat. No. Primers: Qiagen RT² qPCR Primer Assays (PPH) or Qiagen QuantiTect Primer Assays (QT)

**Table S2**

Primer sequences, annealing temperatures and cycles used for RT-PCR analyses in hiPSC pluripotency characterization.

| **Primer** | **Primer for** | **Primer rev** | **Length bps** | **Temp /Cycles** |
| --- | --- | --- | --- | --- |
| OCT4 | GACAACAATGAAAATCTTCAGGAGA | TTCTGGCGCCGGTTACAGAACCA | 218 | 58°C / 36 cycles |
| SOX2 | ATGCACCGCTACGACGTGA | CTTTTGCACCCCTCCCATTT | 437 | 58°C / 30 cycles |
| NANOG | AGTCCCAAAGGCAAACAACCCACTTC | ATCTGCTGGAGGCTGAGGTATTTCTGTCTC | 164 | 64°C / 36 cycles |
| LIN28 | AGTAAGCTGCACATGGAAGG | ATTGTGGCTCAATTCTGTGC | 410 | 58°C / 36 cycles |
| FOXD3 | GTGAAGCCGCCTTACTCGTAC | CCGAAGCTCTGCATCATGAG | 353 | 58°C / 38 cycles |
| GDF3 | TTCGCTTTCTCCCAGACCAAGGTTTC | TACATCCAGCAGGTTGAAGTGAACAGCACC | 311 | 58°C / 32 cycles |
| GAPDH | AGAGGCAGGGATGATGTTCT | TCTGCTGATGCCCCCATGTT | 258 | 58°C / 30 cycles |

**Table S3**

Antibodies and dilutions used for immunocytochemistry of iPSC pluripotency characterization.

| **Primary antibody** | **Type** | **Dilution** | **Supplier** |
| --- | --- | --- | --- |
| hOCT3/4 | polyclonal goat IgG | 1:40 | R&D Systems, #AF1759 |
| hSOX2 | monoclonal mouse IgG1 | 1:200 | Thermo Fisher Scientific, #MA1-014 |
| hNANOG | polyclonal rabbit IgG | 1:100 | Thermo Fisher Scientific, #PA1-097 |
| hLIN28 | polyclonal goat IgG | 1:300 | R&D Systems, #AF3757 |
| hSSEA4 | monoclonal mouse IgG3 | 1:100 | Thermo Fisher Scientific, #MA1-021 |
| hTRA-1-60 | monoclonal mouse IgM | 1:200 | Abcam, #ab16288 |
| hAFP | polyclonal rabbit IgG | 1:100 | DAKO, #A0008 |
| α-SMA | monoclonal mouse IgG2a | 1:3000 | Sigma-Aldrich, #A2547 |
| β-III-Tubulin | monoclonal mouse IgG2A | 1:2000 | Covance, #MMS-435P |
| **Secondary antibody** | **Type** | **Dilution** | **Supplier** |
| Alexa Fluor 488 | polyclonal donkey α-mouse IgG | 1:1000 | Thermo Fisher Scientific, #A21202 |
| Alexa Fluor 555 | polyclonal donkey α-goat IgG | 1:1000 | Thermo Fisher Scientific, #A21432 |
| Alexa Fluor 555 | polyclonal donkey α-mouse IgG | 1:1000 | Thermo Fisher Scientific, #A31570 |
| Alexa Fluor 555 | polyclonal donkey α-rabbit IgG | 1:1000 | Thermo Fisher Scientific, #A31572 |
| FITC | polyclonal goat α-mouse IgM | 1:200 | Jackson Immuno, #115-097-020 |
| **Flow Cytometry** |  | **Dilution** | **Supplier** |
| Alexa Fluor 488 mouse anti-human TRA-1-60 | | 1:50 | BD Biosciences, #560173 |
| Alexa Fluor 647 mouse anti-OCT3/4 | | 1:50 | BD Biosciences, #560329 |

**Figure legends**

**Figure S1 Changes of gene expression levels of pluripotency and cardiac markers as well as ion channels in BrS hiPSC-CMs.** (A) The mRNA levels of the pluripotency gene POU5F1 decrease in hiPSC cells from the 3 donors and the BrS-patient during differentiation to hiPSC-CMs over 35 days. At days 10, 20, 25 and 35 all expression levels are p < 0.05 versus d0 (one way ANOVA with post-tests). (B) The cardiac marker TNNT2 increases during differentiation to hiPSC-CMs over 35 days. At days 10, 20, 25 and 35 all expression levels are p < 0.05 versus d0 (one way ANOVA with post-tests). (C) Immunostaining presents the expression of cardiac specific markers (TNNT, alpha-actinin and Myl4) (D) The mRNA levels of ion channels in hiPSC-CMs from donors and the BrS-patient (*** p < 0.0001, BrS versus donor cells, one way ANOVA with post-tests). (E) The mRNA levels of adrenoceptors and cholinergic receptors in hiPSC-CMs from donors and the BrS-patient (*** p < 0.0001, BrS versus donor cells, one way ANOVA with post-tests). D1, D2, D3 are cells from healthy donors, BrS represents cells from the patient with BrS.

**Figure S2 Late sodium channel currents and sodium channel protein expression levels in hiPSC-CMs.** Sodium channel currents (I_Na_) were recorded using the indicated protocol in D and the late I_Na_ at 350 ms of the depolarization pulse was analyzed. TTX (30 µM) was applied to identify the sodium current and the TTX-sensitive late currents were valuated as late I_Na_. (A) Western blots showing the expression of SCN5A and SCN1B proteins. (B) Averaged values (normalized to GAPDH as housekeeping gene) of pixel density from western blots showing the expressions of SCN5A and SCN1B proteins. (C) Averaged values of late I_Na_ in donor (D1, D2, D3) and BrS cells. (D) Representative traces of late I_Na_ from 350 to 360 ms of depolarization pulses.

**Figure S3 Calcium channel currents were not changed in hiPSC-CMs from the BrS patient.** The L-type calcium channel currents (I_Ca_) were evoked by the protocol shown in B (inset) and were plotted against the test potentials to obtain I-V curves. I_Ca_ was divided by voltage (the driving force for I_Ca_) to obtain the conductance (Gm). Gm was normalized to Gmax and plotted against the voltages to get the activation curves. For assessing the inactivation of Ca channels, the currents measured with the protocol shown in F (inset) was plotted against voltages to get the inactivation curves. The activation and inactivation curves were fitted by Boltzmann equation and the voltages of half maximal (V0.5) activation or inactivation were obtained. For assessing the recovery from inactivation, double pulses (inset in H) were used. The currents induced by the second pulse were normalized to that induced by the first pulse and plotted against the time intervals between both pulses and the time constants were obtained by exponential fitting. All the parameters of I_Ca_ were compared between a donor (D1, D2, D3) and the BrS patient. (A) Representative traces of I_Ca_ in donor (D1, D2 and D3) and BrS cells. (B) I-V curves of peak I_Ca_. (C) Mean values of peak I_Ca_ at -10 mV. (D) Activation curves of peak I_Ca_. (E) Mean values of V0.5 of activation. (F) Inactivation curves of peak I_Ca_. (G) Mean values of V0.5 of inactivation. (H) Time course curves of recovery from inactivation of peak I_Ca_. (I) Mean values of time constants (tau) of recovery of I_Ca_. n, number of cells; n.s., not statistically significant (p>0.05).

**Figure S4 Intracellular calcium concentration in donor and BrS hiPSC-CMs.** Calcium transients were recorded in BrS-cells loaded with Fluo-3 and compared with that recorded in donor (D1, D2) cells. (A) Representative traces of calcium transients in donor (D1, D2) and BrS cells. (B) Mean values of systolic calcium concentrations in donor (D1, D2) and BrS cells. (C) Mean values of diastolic calcium concentrations in donor (D1, D2) and BrS cells. Values shown are mean ± SEM. n, number of cells. n.s., not statistically significant (p>0.05).

**Figure S5 Potassium channel currents in hiPSC-CMs.** Potassium channel currents recorded using different protocols (insets in B, E, H). 4-AP (3 mM), E-4031 (3 µM) and chromanol 293 (10 µM) were used to separate I_to_, I_Kr_ and I_Ks_, respectively. The blocker-sensitive currents were analyzed. (A) Representative traces of I_to_ in donor (D1,D2) and BrS cells. (B) I-V curves of peak I_to_ in donor and BrS cells. (C) Mean values of peak I_to_ at 60 mV. (D) Representative traces of I_Kr_ in donor (D1,D2) and BrS cells. (E) I-V curves of I_Kr_ in donor and BrS cells. (F) Mean values of I_Kr_ at 30 mV. (G) Representative traces of I_Ks_ in donor (D1,D2) and BrS cells. (H) I-V curves of I_Ks_ in donor and BrS cells. (I) Mean values of I_Ks_ at 30 mV. Values shown are mean ± SEM. n, number of cells; n.s., not statistically significant (p>0.05).

**
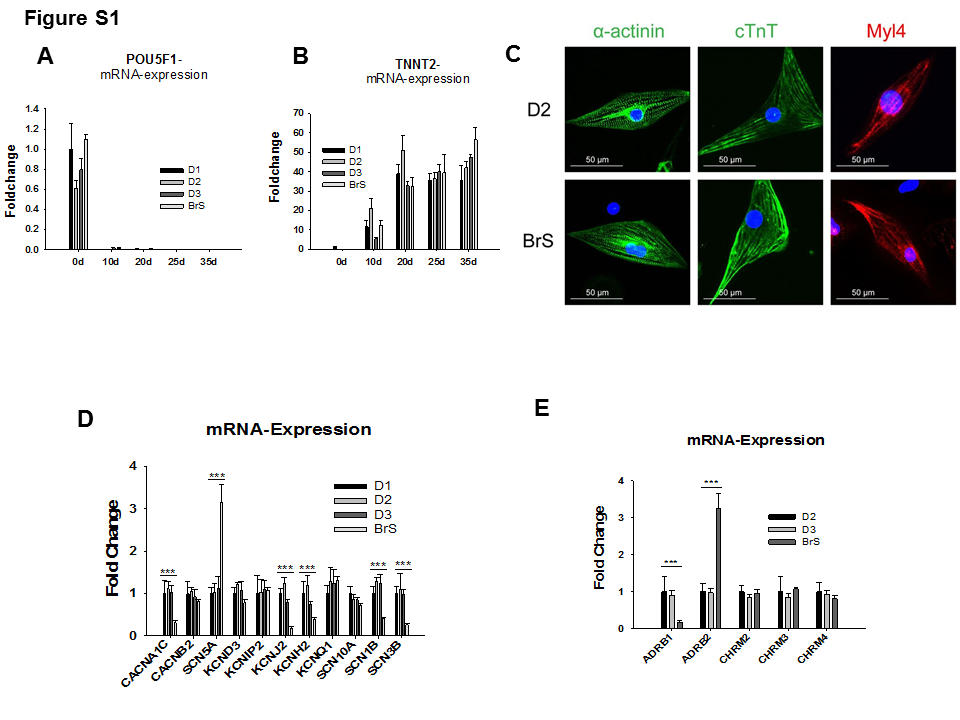
**

**
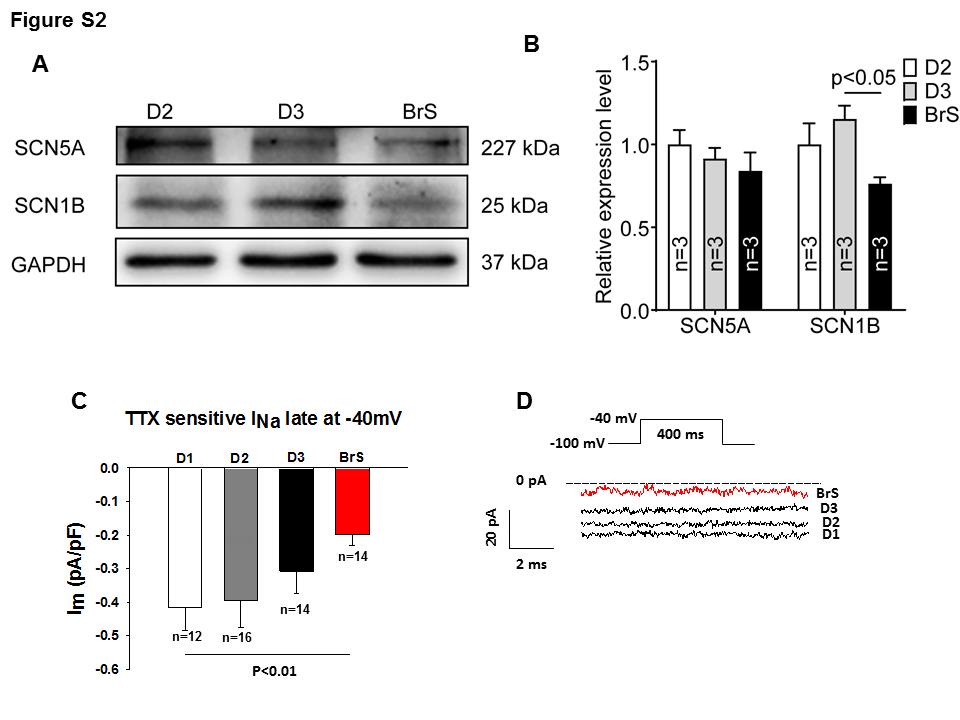
**

**
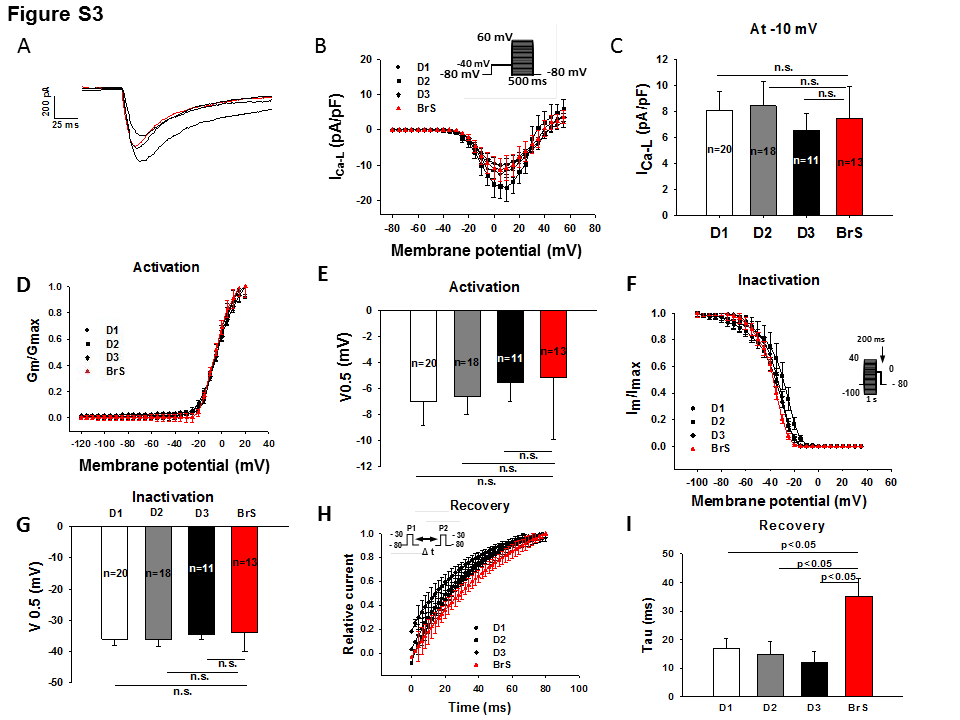
**

**
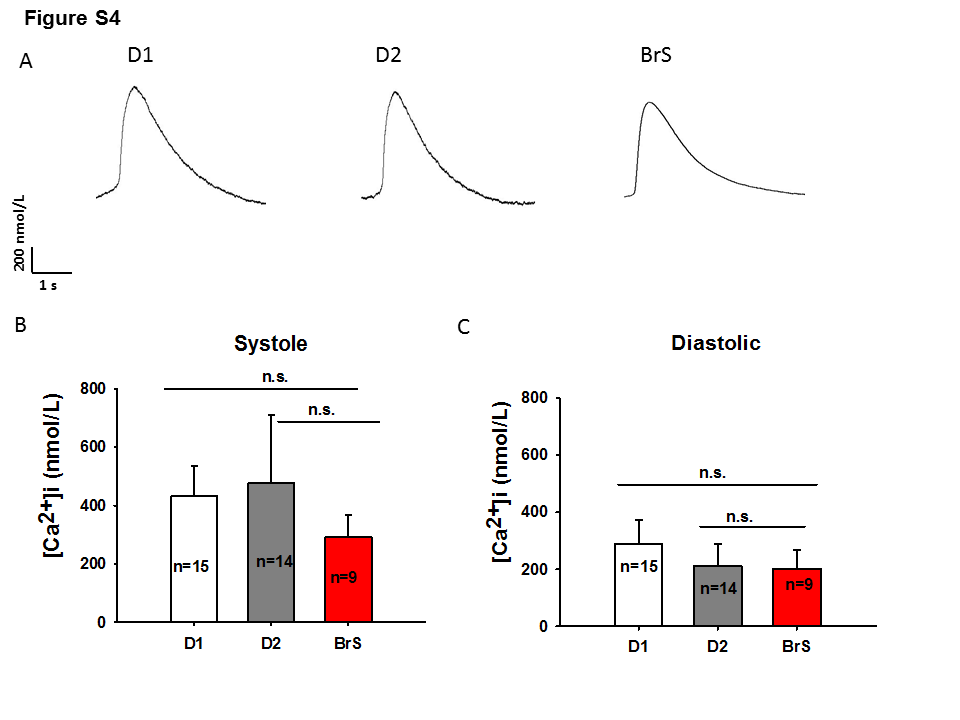
**

**
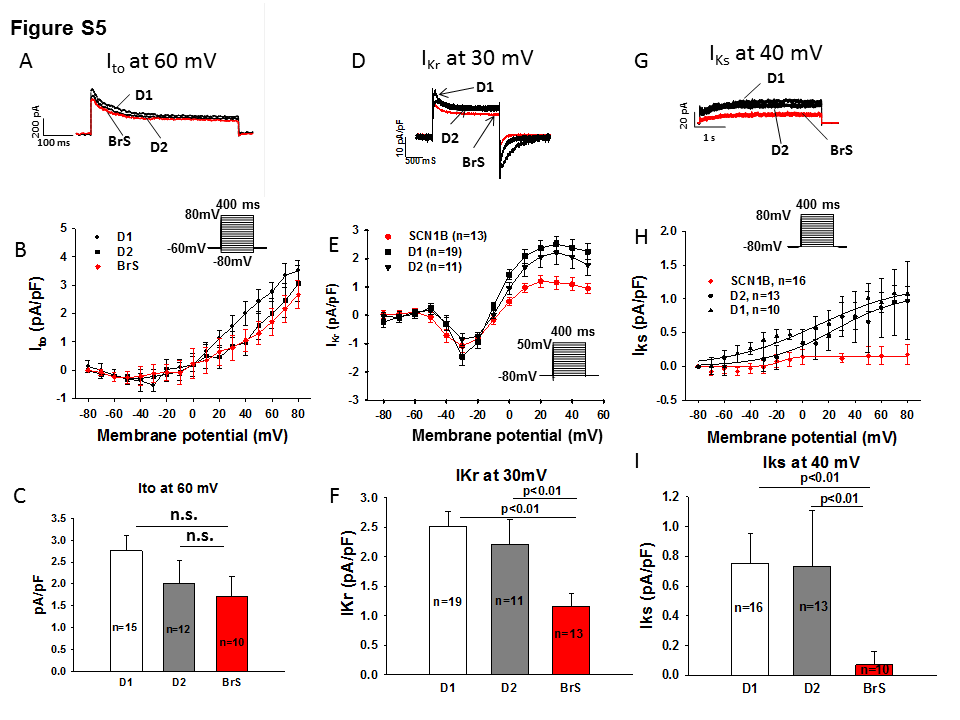
**
